# Perceived risk and protective behaviors regarding COVID-19 among Iranian pregnant women

**DOI:** 10.1186/s43043-020-00038-z

**Published:** 2020-09-18

**Authors:** Soodabeh Aghababaei, Saeed Bashirian, Alireza Soltanian, Mansoureh Refaei, Tahereh Omidi, Samereh Ghelichkhani, Farzaneh Soltani

**Affiliations:** 1 Mother and Child Care Research Center, Hamadan University of Medical Sciences, Hamadan, Iran; 2 Department of Public Health, School of Health, Social Determinants of Health Research Center, Hamadan University of Medical Sciences, Hamadan, Iran; 3 Department of Biostatistics, School of Public Health, Hamadan University of Medical Sciences, Hamadan, Iran; 4 Student Research Committee, Hamadan University of Medical Sciences, Hamadan, Iran; 5 Deputy of Treatment, Hamadan University of Medical Sciences, Hamadan, Iran

**Keywords:** COVID-19, Health behavior, Knowledge, Pregnant women, Protective, Risk

## Abstract

**Background:**

Despite the vulnerability of pregnant women, few studies have been conducted on their perceived risk and protective behaviors during the COVID-19 pandemic. The present cross-sectional study aims to investigate the perceived risk and protective behaviors regarding COVID-19 among pregnant women, in Hamadan, Iran. Using a two-stage cluster sampling method, 225 pregnant women referring to the health centers completed the questionnaires. Data were analyzed using the Kruskal-Wallis and Spearman correlation tests as well as a stepwise linear regression model at 95% confidence level.

**Results:**

93.8% of pregnant women had a high level of knowledge, 97.3% had a high performance in protective behaviors, and 72.9% had a moderate level of risk perception related to COVID-19. The highest mean score of knowledge was observed in women who had a history of influenza in their previous pregnancies (90.97 ± 5.94). The mean score of protective behaviors was significantly higher in women with a high economic level (97.78 ± 5.11), and the highest level of risk perception was observed in nulliparous women (59.97 ± 9.80). Risk perception was an independent predictor of protective behaviors related to COVID-19 (*P* < 0.05).

**Conclusions:**

Pregnant women had a high level of knowledge, high performance in protective behaviors, and a moderate level of risk perception related to COVID-19. History of influenza in previous pregnancies, high economic level, and nulliparity were associated with higher levels of knowledge, protective behaviors, and risk perception, respectively. Risk perception of pregnant women regarding COVID-19 can predict their protective behaviors.

## Background

In the last days of 2019, an unknown outbreak of pneumonia was reported in eastern China, which was recognized by the World Health Organization as COVID-19 [1]. The most important route of transmission agreed by all is respiratory droplets or direct human-to-human contact [2–4]. COVID-19, which has now become a pervasive epidemic, is a serious public health emergency that is deadly, especially in vulnerable populations and communities where health systems are not sufficiently prepared to manage the infection [4].

Pregnant women and their fetuses are at high risk during the outbreak of infectious diseases [5]. In general, physiological and mechanical changes during pregnancy increase susceptibility to infections. Researchers have shown that emerging infections have a significant impact on pregnant women and their fetuses [6]. Previous data from SARS and MERS infections show that clinical findings during pregnancy can range from mild symptoms to severe illness and death [7]. It seems that coronavirus infection in the third trimester of pregnancy increases the risk of premature rupture of the membranes, preterm labor, fetal tachycardia, and fetal distress [8–10]. Therefore, it is important that pregnant women and their families, as well as health care providers, receive as much accurate information as possible. During the corona epidemic, it is recommended that pregnant women take care of personal and social hygiene and avoid unnecessary trips, crowded places, public transportation, and contact with sick people [9]. Pregnant women have different attitudes toward the recommendations of health care providers. Some evaluate the given instructions based on their views and experiences and change them if they do not feel useful, but some women carefully follow the treatment plans and welcome health care to reduce the risk [11].

In pandemic diseases, perceived risk encourages people to engage in protective behaviors to reduce potential risks [12]. Globally, risk is defined as the probability of loss and is thought to be composed of two components: probability and severity of negative outcomes [13, 14]. Meanwhile, risk perception is defined as a person’s expectation of a particular event occurring [15]. Risk perception as an important determinant of healthy behaviors plays an important role in designing interventions to change behaviors [16]. In the study of Ackerman et al. on the Iranian pregnant women, the higher fear of COVID-19 was associated with higher preventive behaviors. COVID-19 pandemic is the most intense and emotional experience of pregnant women’s lives. In this regard, health care providers should pay more attention to pregnant women who have health concerns at the same time about themselves and their fetuses. Currently, the mortality rate from COVID-19 is lower than previous epidemics such as SARS and MERS. However, stress and anxiety may be the most important challenge in the COVID-19 pandemic [17]. Transmitting large amounts of information and overuse of the media can lead to overreaction, irrational fear, and over-perception of risk, which in turn might affect pregnant women’s behaviors [18]. Psychobehavioral surveillance is critical during communicable disease outbreaks because it affects risk awareness strategies targeting high-risk groups such as pregnant women [19]. Measuring the level of risk perception as well as its determinants in pregnant women is essential for the transmission of information and health protocols. Despite the vulnerability of pregnant women during the COVID-19 pandemic, few studies have been conducted on their risk perception and protective behaviors. The present study aims to investigate the perceived risk and protective behaviors regarding COVID-19 among Iranian pregnant women.

## Methods

### Participants and study design

This cross-sectional study was conducted on 225 pregnant women referring to the health centers to receive prenatal care in Hamadan, Iran, 2020. The inclusion criteria included normal singleton pregnancies, without medical or obstetrical complications, and the ability to read and write. Pregnant women who did not answer more than 10% of the questionnaire items were excluded from the study.

### Sampling

Considering the standard deviation of the knowledge of COVID-19 in women (about 16.58), 96% confidence level, and maximum error equal to 15% standard deviation, the sample size was calculated to be 225 pregnant women. The sample was drawn through a two-stage cluster sampling method. First, Hamadan city was divided into four geographical regions (north, south, east, and west), and then two health centers were randomly selected from each region using the list of health centers (8 health centers in total). Thirty eligible pregnant women were randomly selected from each center, and after obtaining the informed consent of the participants, the self-administered questionnaires were completed with observing health protocols.

### Instruments

The questionnaire consisted of 4 sections including the following:

1. Socio-demographic and obstetrics characteristics: Including age, education level, place of residence, housing status, economic situation, gestational age, parity, pregnancy status, history of abortion, number of children, history of influenza in the previous pregnancies, and sources of information about COVID-19.
2. COVID-19-related knowledge (16 items): The items were designed based on the health protocols by the World Health Organization [20], as well as the Ministry of Health and Medical Education, Iran (Table 1). The aspects of knowledge included etiology, symptoms, transmission, and public prevention. Correct answers were assigned 1 point, and incorrect answers or “I do not know” were assigned 0 points. The total score was converted into a percentile. A score ≥ 75% was designated as high, 50–75% as moderate, and ≤ 50% as low level of knowledge.
3. COVID-19-related risk perception (7 items): Using the visual scale measure [21, 22], perceived risks of maternal infection, fetal infection, fetal anomalies, abortion or fetal death, preterm delivery, hospitalization the newborn in the neonatal intensive care unit, and maternal death due to coronavirus infection were measured. Responses were given based on a Lickert range from 0 (no perceived risk) to 10 (high level of perceived risk). The total score, which was between 0 and 70, was calculated from the score of 100. In this study, a score of less than 40 was considered the low perceived risk, a score between 40 and 60 was considered the moderate perceived risk, and a score higher than 60 was considered the high perceived risk.
4. COVID-19-related protective behaviors (11 items): The items were designed based on the health protocols by the World Health Organization [20], as well as the Ministry of Health and Medical Education, Iran (Table 1). The aspects of preventive behaviors included social distancing, personal hygiene and frequent hand washing, public transportation, out-of-home prepared foods, and shopping. Responses were “yes” or “no,” and the participant was assigned 1 point for each appropriate behavior and 0 point for inappropriate behaviors. The total score was converted into a percentile. A score ≥ 75% was designated as high, 50–75% as moderate, and ≤ 50% as low level of knowledge.

**Table 1:** Distribution of participants’ responses to the items of knowledge and protective behaviors

COVID-19-related knowledge		
Questions	Items (true, false, and I do not know)	Correct answer rate (0–100%)
Q01	Corona disease is an infectious caused by the coronavirus.	**90.1**
Q02	The first case of the disease was diagnosed in China.	**96**
Q03	The origin of the disease is unknown, but it appears to have been transmitted to humans by bats, seafood or snakes.	**88.8**
Q04	Common symptoms of the disease are fever, cough, and shortness of breath.	**98.2**
Q05	A person infected with the virus may look completely healthy.	**94.7**
Q06	If infected, the person should quarantine for 14 days.	**98.2**
Q07	Transmission is through respiratory droplets such as cough and sneezing.	**95.6**
Q08	The disease is transmitted through close contact with an infected person.	**96.4**
Q09	Children do not get the disease.	**68**
Q10	Pregnant women are more susceptible to the disease.	**96**
Q11	The disease can be prevented by hand washing and personal hygiene.	**96.9**
Q12	Medical masks are useful to prevent the transmission of respiratory drops.	**96**
Q13	Lack of close contact can prevent the onset of the disease.	**94.7**
Q14	Everyone in the community should wear a mask.	**28.9**
Q15	Coronavirus can be treated with common antiviral drugs.	**59.1**
Q16	The flu vaccine can be given every year to prevent coronavirus.	**55.1**
**COVID-19-related protective behaviors**		
Questions	Items (yes or no)	% of yes
Q01	I have canceled meetings with family and friends.	**92.9**
Q02	I do not eat out-of-home prepared foods or in restaurants.	**96**
Q03	I canceled going to the barber shop.	**96**
Q04	I have reduced the use of public transportation.	**98.2**
Q05	I do not go out shopping.	**89.9**
Q06	I have reduced going to closed spaces such as libraries, theaters, and cinemas.	**99.6**
Q07	I avoid coughing near people, as much as possible.	**99.1**
Q08	I have avoided places where a large number of people have gathered.	**99.1**
Q09	I have increased the cleaning and disinfection of items that can be easily touched by hand (i.e., door handles and surfaces).	**96.4**
Q10	I get my hands washed more than usual.	**92**
Q11	I avoid hugging and kissing children and family members.	**92.9**
Q12	I keep my distance from others at home.	**89.3**
Q13	I clean and disinfect items that are purchased from abroad.	**98.2**
Q14	My family and I watch TV programs that teach the basics of health care-related.	**98.7**
Q15	I have talked to my family and friends about preventing coronavirus.	**98.7**

The validity of the questionnaire was assessed using different experts’ opinions, and its reliability was calculated using the Cronbach *α* coefficient (0.89).

### Statistical analysis

Statistical analysis was performed using SPSS/24.0, at the 5% significance level. Using the Kolmogorov-Smirnov test, none of the major outcomes followed a normal distribution and was analyzed using the Kruskal-Wallis and Spearman correlation tests. Stepwise multivariate linear regression analysis was performed to determine the most predictive indicator for preventive behaviors. The frequencies and percentages were computed for categorical variables, and the means and standard deviations were calculated for numerical variables.

## Results

A total of 240 pregnant women were enrolled in this study, and 225 people responded to the questionnaires. The mean age of women was 30.24 years. The majority of participants had college education (36.4%), were unemployed (90.2%), had a moderate economic situation (72.9%), and lived in their personal houses (49.8%). The mean gestational age was 25.71 weeks. The majority of participants were primigravida (45.8%), had a wanted pregnancy (79.6%), had regular prenatal care (95.6%), and did not have a history of abortion (78.2%) or influenza in the previous pregnancy (96%); 71.1% of pregnant women said that television was the main source of their information about COVID-19.

Table 1 shows the distribution of participants’ responses to the items of knowledge and preventive behaviors related to COVID-19. The average of correct answers was 84.54% in the knowledge section. The lowest scores pertained to Q9 (children do not get the disease), Q14 (everyone in the community should wear a mask), Q15 (coronavirus can be treated with common antiviral drugs), Q16 (the flu vaccine can be given every year to prevent the coronavirus). In the preventive behaviors section, the average of correct answers was 95.8%. The lowest score (89.3%) pertained to “I keep my distance from others at home.” According to the results, 93.8% of pregnant women had a high level of knowledge related to COVID-19. 97.3% of the participants had high performance in preventive behaviors, and 72.9% of pregnant women had a moderate level of risk perception related to the disease (Table 2). The mean score of knowledge in women who had a university education, their source of information was an obstetrician, had two or more children, and had a history of influenza in the previous pregnancy was significantly higher (*P* < 0.05) (Table 3).

**Table 2:** COVID-19-related knowledge, protective behaviors, and risk perception among pregnant women

Variable	Level, no. (%)	Mean ± SD (range 0–100)
Knowledge		
High	211 (93.8)	85.72 ± 7.39
Moderate	13 (5.8)	
Low	1 (0.4)	
Protective behaviors		
High	219 (97.3)	95.79 ± 7.05
Moderate	6 (2.7)	
Low	–	
Risk perception		
High	27 (12)	48.07 ± 10.25
Moderate	164 (72.9)	
Low	34 (15.1)

**Table 3:** Differences in major variables according to demographic and obstetrics characteristics

Variable	No. (%)	Knowledge, mean ± SD, test statistics^ab^	Protective behaviors, mean ± SD, test statistics^ab^	Risk perception, mean ± SD, test statistics^ab^
Age (years)				
15–25	54 (24)	84.38 ± 8.37	94.57 ± 9.34	47.46 ± 10.61
26–36	140 (62.2)	86.21 ± 6.69	96.19 ± 6.15	48.34 ± 10.02
37–47	31 (13.8)	85.89 ± 8.35	96.13 ± 5.91	47.93 ± 10.90
		2.424	0.426	0.862
Gestational age				
1–12	22 (10.0)	83.95 ± 10.53	94.55 ± 7.60	49.03 ± 8.65
13–24	68 (30.8)	85.29 ± 6.89	96.08 ± 6.33	49.04 ± 10.29
25–36	131 (59.3)	86.24 ± 7.12	95.83 ± 7.14	47.41 ± 10.55
		1.749	1.047	2.523
Education				
High school	66 (29.3)	**84.94 ± 9.19**	95.25 ± 7.09	47.71 ± 11.04
Diploma	77 (34.2)	**84.78 ± 7.64**	96.88 ± 6.54	47.99 ± 10.13
Collegiate	82 (36.4)	**87.23 ± 5.01**	95.20 ± 7.43	48.45 ± 9.80
		**8.787**^a^*	5.258	0.870
Housing status				
Personal	112 (49.8)	86.16 ± 7.07	95.95 ± 7.18	47.46 ± 9.99
Rent	107 (47.6)	85.28 ± 7.68	95.64 ± 7.04	48.60 ± 10.59
Others	6 (2.7)	85.42 ± 9.20	95.56 ± 5.44	50.00 ± 9.56
		0.731	0.691	0.349
Economic situation				
Weak	43 (19.1)	85.17 ± 8.44	**94.57 ± 6.38**	47.08 **±** 10.77
Moderate	164 (72.9)	85.50 ± 7.25	**95.89 ± 7.36**	48.24 ± 10.23
Good	18 (8.0)	89.06 ± 5.28	**97.78 ± 5.11**	48.98 ± 9.49
		4.514	**6.339**^a^*	0.602
Information source				
Obstetrics	16 (7.1)	**88.87 ± 5.35**	94.58 ± 9.50	50.98 ± 9.44
Midwives	27 (12)	**88.43 ± 7.08**	95.06 ± 6.02	44.19 ± 8.27
TV	160 (71.1)	**84.80 ± 7.76**	96.42 ± 6.43	48.57 ± 10.53
Others	22 (9.8)	**86.79 ± 4.52**	93.03 ± 9.75	47.14 ± 9.37
		**8.787**^a^*	5.258	6.042
Parity				
0	88 (39.1)	85.37 ± 6.11	96.29 ± 6.62	**59.97 ± 9.80**
1	87 (38.7)	85.17 ± 8.73	95.71 ± 7.19	**48.69 ± 10.68**
2	42 (18.7)	87.28 ± 6.88	94.44 ± 7.29	**47.70 ± 10.60**
3+	8 (3.6)	87.50c7.09	98.33 ± 4.71	**43.46 ± 10.60**
		0.628	3.138	**6.510**^a^*
Number of abortion				
0	176 (78.2)	85.44 ± 7.48	95.68 ± 7.06	**58.38 ± 10.39**
1	44 (19.6)	86.58 ± 7.34	96.67 ± 6.97	**47.47 ± 9.85**
2+	5 (2.2)	88.13 ± 4.64	92 ± 7.30	**42.54 ± 8.28**
		1.167	0.092	**4.208**^a^*
Number of children				
0	104 (46.2)	**85.46 ± 6.44**	96.09 ± 6.67	48.39 ± 10.12
1	88 (39.1)	**84.69 ± 8.53**	95.15 ± 7.81	48.18 ± 10.64
2+	33 (14.7)	**89.3 ± 5.59**	96.57 ± 6.04	46.80 ± 9.79
		**7.126**^a^*	1.046	0.286
Pregnancy status				
Wanted	9 (4)	85.75 ± 7.24	98.52 ± 2.94	45.56 ± 13.08
Unwanted	216 (96)	85.60 ± 8.08	95.68 ± 7.15	48.18 ± 10.14
		3912.112	859.010	764.500
History of influenza				
Yes	179 (79.6)	**90.97 ± 5.94**	95.83 ± 7.31	47.56 ± 10.18
No	46 (20.4)	**85.50 ± 7.38**	95.65 ± 6.00	50.08 ± 10.39
		**546.200**^b*^	3779.001	3578.532

*M* mean, *SD* standard deviation

^a^Kruskal-Wallis value

^b^Mann-Whitney *U* value

*Significant at the level of *P* < 0.05

The highest mean score of knowledge was observed in women who had a history of influenza in their previous pregnancies (90.97 ± 5.94), and even in these women, the average level of risk perception and preventive behavior was higher than in pregnant women who did not have a history of influenza. The mean score of preventive behaviors was significantly higher in women with a high economic level (*P* < 0.05), so the highest score of preventive behaviors (97.78 ± 5.11) also belonged to this group of women. The mean score of risk perception in nulliparous women and those without a history of abortion was significantly higher (*P* < 0.05). The highest level of risk perception was observed in nulliparous women (59.97 ± 9.80), and the lowest level was observed in pregnant women who experienced two or more abortions (42.54 ± 8.28).

The Spearman correlations between knowledge, protective behaviors, and risk perception related to COVID-19 are presented in Table 4. Risk perception was positively associated with protective behaviors (*r* = 0.146, *P* = 0.031). The knowledge negatively associated with protective behaviors, but this relationship was not statistically significant (*r* = − 0.125, *P* = 0.069). We also performed a linear regression analysis considering all studied risk factors to determine the most predictive indicator for protective behaviors. In Table 5, we present the final multivariate linear regression model. Stepwise multivariate linear regression analysis revealed that risk perception was an independent predictor of protective behaviors (*β* = 0.146, *P* = 0.029).

**Table 4:** Correlations between COVID-19-related knowledge, protective behaviors, and risk perception among pregnant women

Variables	Knowledge	Protective behavior	Risk perception
Knowledge	1	–	–
Protective behaviors	− 0.125	1	–
Risk perception	0.016	0.146*	1

*Significant at the level of *P* < 0.05; computed by Spearman rank correlation

**Table 5:** Multivariate regression analysis with protective behaviors as a dependent variable

Independent variables	***B***	SE	Beta	***T***	***P***
Risk perception	0.340	0.154	0.146	2.199	0.029*
Knowledge	− 0.091	0.124	− 0.049	− 0.734	0.464

*Significant at the level of *P* < 0.05

## Discussion

Although the effect of COVID-19 on pregnant women is still unclear, there are concerns about its potential impact on maternal and perinatal outcomes due to suppression of the immune system during pregnancy [23]. However, few studies have been conducted on perceived risk and protective behaviors among pregnant women during the COVID-19 pandemic. The present study addressed this important issue and measured the level of risk perception, knowledge level, and protective behaviors of pregnant women, as well as the relevant determinants.

The present study showed that the level of knowledge related to the COVID-19 and its transmission and prevention was high among pregnant women, so this level of awareness about the symptoms of the disease, and in particular the ways in which it is transmitted, has been almost above 95%. It seems that the awareness of pregnant women, both through the mass media and by health care providers, has increased significantly. In a study by Nwafor et al., 60.9% of pregnant women had sufficient knowledge of preventive measures against COVID-19 [24]. Yassa et al., in their study on pregnant women near childbirth, found that pregnant women have a positive attitude toward quarantine. At the same time, they expressed their progressive anxiety and concern for the pregnancy and the baby due to the pandemic and also believed that they had been given insufficient counseling or limited information about the relationship between pregnancy and pandemic [25].

In the present study, the majority of pregnant women [about 62%] believed that the general population should use masks to prevent disease; nearly 29% opposed it, and about 10% said they were unaware. It should be acknowledged that the unknown nature of COVID-19, and even its transmission, along with the different policies of different countries on disease protection, can have a significant impact on people’s responses. Besides, researchers have noted the sensitivity and concern of pregnant women about their vulnerability to infectious diseases. For example, an interview study conducted during the H1N12009 Influenza pandemic revealed that the individuals most concerned for the possibility of getting infected or transmitting the virus to others were pregnant women and those with young children [26, 27]. In the present study, 32% of pregnant women did not know that children could also develop COVID-19. About 40% of women did not know that the disease could not be treated with common antiviral drugs, and about 45% of pregnant women thought that the flu vaccine could be given every year to prevent the COVID-19. While the clinical evidence was growing rapidly, this data may guide to perceive what accurate information should be provided to pregnant women.

Our results showed that more than half of pregnant women obtained their information through TV. Similar to our findings, other studies reported that participants usually obtained their information about infectious diseases through the internet and watching TV. Olapegba and Ayandele in a study in Nigeria reported that traditional media [TV/radio] was the source of information regarding COVID-19 for more than 93.5% of people [28]. In a similar manner, Sasaki et al. found that television, the Internet, and newspapers were the most common sources of information about the H1N1 outbreak [29]. According to our study, women with a university education were significantly more aware of the disease than women with less education. In the recent study of Nwafor et al., one of the factors associated with inadequate knowledge of preventive measures regarding COVID-19 was no formal education [24]. The level of knowledge of pregnant women who had more children was significantly higher, and although nonsignificant, their risk perception was lower and their preventive behavior was better. Contrary to our study, in the study of Nwafor et al., pregnant African women who had given birth five or more times had lower levels of awareness about preventive measures related to COVID-19 [24]. The greater awareness of pregnant women with a previous history of influenza in the present study can be related to the increased sensitivity of this group of women to the risk of contracting viral diseases during pregnancy and its complications.

Pregnant women in our study reported high levels of protective behaviors related to COVID-19. It seems that during acute conditions such as epidemics, due to extensive training and information transition, high preventive behavior can be expected from individuals. In the USA, over the course of a few days, people became increasingly aware of the dangers of COVID-19 and performed well on protective behaviors [30]. In our study, pregnant women with better economic status had better protective behaviors. In a study by Chandrasekaran et al. [31], knowledge and behavior related to Zika disease were lower in women with poor economic status compared to women with moderate to high status. Therefore, special attention should be paid to women with low economic status, especially during pandemics.

Nulliparous women in our study had a higher level of risk perception related to COVID-19 than multiparous women. Similar to the risk perception in other fields, pregnancy risk perception is highly individualized and several factors may influence the perception of pregnancy risk [17]. Risk perception is the subjective response based on previous life experiences, coping strategies, the context in which the risk occurs, and the degrees the risk obtained from a variety of sources [32]. Little is known about how risk impacts a woman’s perception and experience of pregnancy [33]. However, it seems that the experience of pregnancy and childbirth can reduce the perceived risk.

The results of the present study showed that risk perception can predict preventive behaviors significantly. In contrast to our study, Taghrir et al., in the recent study on the medical students, reported a negative correlation between preventative behaviors and risk perception related to COVID-19 [34]. Risk perception as a determinant of protective behaviors is often positively associated with preventative behaviors, although in some cases negative interactions with preventive behaviors have been shown, for example, when the perception of risk is high, but the chance of success in dealing with it is considered low, preventive behaviors are reduced [35].

### Limitations

The main strong point of the present study is addressing pregnant women as a vulnerable group during the COVID-19 pandemic. Yet, the causal relationships among the variables could not be assessed due to the cross-sectional design of the study.

## Conclusions

Iranian pregnant women had a high level of knowledge, high performance in protective behaviors, and a moderate level of risk perception related to COVID-19. History of influenza in previous pregnancies, high economic level, and nulliparity were associated with higher levels of knowledge, protective behaviors, and risk perception, respectively. Risk perception of pregnant women can predict their protective behaviors against COVID-19. The results of the present study, as one of the first study on the risk perception and protective behaviors of Iranian pregnant women during the COVID-19 pandemic, can be used by researchers and health planners in similar future crises.

## Data Availability

The datasets used and/or analyzed during the current study are available from the corresponding author on reasonable request.

## Abbreviation

COVID-19: Coronavirus disease of 2019

## References

[1] Zhu N, Zhang D, Wang W, Li X, Yang B, Song J (2010). A novel coronavirus from patients with pneumonia in China, 2019. N Engl J Med.

[2] Rothe C, Schunk M, Sothmann P (2020). Transmission of 2019-nCoV infection from an asymptomatic contact in Germany. N Engl J Med.

[3] Chen NS, Zhou M, Dong X (2020). Epidemiological and clinical characteristics of 99 cases of 2019 novel coronavirus pneumonia in Wuhan, China: a descriptive study. Lancet.

[4] McIntosh K. Coronavirus disease 2019 (COVID-19). Available from: https://www.uptodate.com/contents/126981. Updated February 24, 2020.

[5] Dashraath P, Jeslyn WJL, Karen LMX, Min LL, Sarah L, Biswas A (2020). Coronavirus disease 2019 (COVID-19) pandemic and pregnancy. Am J Obstet Gynecol.

[6] Rasmussen SA, Hayes EB (2005). Public health approach to emerging infections among pregnant women. Am J Public Health.

[7] Rasmussen SA, Smulian JC, Lednicky JA, Wen TS, Jamieson DJ (2020). Coronavirus disease 2019 (COVID-19) and pregnancy: what obstetricians need to know. Am J Obstet Gynecol.

[8] Liang H, Acharya G (2020). Novel corona virus disease (COVID-19) in pregnancy: what clinical recommendations to follow?. Acta Obstet Gynecol Scand.

[9] Wang SS, Zhou X, Lin XG (2020). Experience of clinical management for pregnant women and newborns with novel coronavirus pneumonia in Tongji Hospital, China. Curr Med Sci.

[10] Zhang L, Jiang Y, Wei M (2020). Analysis of the pregnancy outcomes in pregnant women with COVID-19 in Hubei Province. Zhonghua Fu Chan Ke Za Zhi.

[11] Heaman M, Gupton A, Gregory D (2004). Factors influencing pregnant women’s perceptions of risk. MCN Am J Matern Child Nurs.

[12] Oh SH, Lee SY, Han C (2020) The effects of social media use on preventive behaviors during infectious disease outbreaks: the mediating role of self-relevant emotions and public risk perception [published online ahead of print, 2020 Feb 16]. Health Commun 1-10. 10.1080/10410236.2020.1724639 32064932

[13] Pilarski R (2009). Risk perception among women at risk for hereditary breast and ovarian cancer. J Genet Couns.

[14] Wachinger G, Renn O, Begg C, Kuhlicke C (2013). The risk perception paradox--implications for governance and communication of natural hazards. Risk Anal.

[15] Henrich L, McClure J, Doyle EEH (2018). Perceptions of risk characteristics of earthquakes compared to other hazards and their impact on risk tolerance. Disasters.

[16] Ferrer R, Klein WM (2015). Risk perceptions and health behavior. Curr Opin Psychol.

[17] Bassetti M, Vena A, Giacobbe DR (2020). The novel Chinese coronavirus (2019-nCoV) infections: challenges for fighting the storm. Eur J Clin Invest.

[18] Huynh TLD (2020). Data for understanding the risk perception of COVID-19 from Vietnamese sample. Data Brief.

[19] Wong LP, Sam IC (2011). Behavioral responses to the influenza A (H1N1) outbreak in Malaysia. J Behav Med.

[20] World Health Organization (2020) Coronavirus disease (COVID-19) advice for the public [website]. 2020. https://www.who.int/emergencies/diseases/novel-coronavirus-2019/advice-for-public. Accessed 30 Jan 2020.

[21] Heaman MI, Gupton AL (2009). Psychometric testing of the Perception of Pregnancy Risk Questionnaire. Res Nurs Health.

[22] Taghizadeh Z, Cheraghi MA, Kazemnejad A, Pooralajal J, Aghababaei S (2017). Difference in perception of pregnancy risk in two maternal age groups. J Clin Diagn Res.

[23] Zhou F, Yu T, Du R (2020). Clinical course and risk factors for mortality of adult inpatients with COVID-19 in Wuhan, China: a retrospective cohort study. Lancet.

[24] Nwafor JI, Aniukwu JK, Anozie BO, Ikeotuonye AC, Okedo-Alex IN (2020). pregnant women’s knowledge and practice of preventive measures against COVID-19 in a low-resource African setting. Int J Gynaecol Obstet.

[25] Yassa M, Birol P, Yirmibes C, et al (2020) Near-term pregnant women’s attitude toward, concern about and knowledge of the COVID-19 pandemic. J Matern Fetal Neonatal Med Ahead-of-print: 1-8. Doi:10.1080/14767058.2020.1763947 10.1080/14767058.2020.1763947 32429780

[26] Braunack-Mayer A, Tooher R, Collins JE, Street JM, Marshall H (2013). Understanding the school community’s response to school closures during the H1N1 2009 influenza pandemic. BMC Public Health.

[27] Brooks SK, Webster RK, Smith LE (2020). The psychological impact of quarantine and how to reduce it: rapid review of the evidence. Lancet.

[28] Olapegba PO, Ayandele O (2020). Survey data of COVID-19-related knowledge, risk perceptions and precautionary behavior among Nigerians. Data Brief.

[29] Sasaki TK, Yoshida A, Kotake K (2013). Attitudes about the 2009 H1N1 influenza pandemic among pregnant Japanese women and the use of the Japanese municipality as a source of information. Southeast Asian J Trop Med Public Health.

[30] Wise T, Zbozinek TD, Michelini G, Hagan CC (2020) Changes in risk perception and protective behavior during the first week of the COVID-19 pandemic in the United States. PsyArXiv Preprints. 10.1098/rsos.200742 PMC7540790 33047037

[31] Chandrasekaran N, Marotta M, Taldone S, Singh V, Koru-Sengul T, Curry C (2018). Socioeconomic disparities in knowledge and behaviors of Zika virus in pregnant women [6J]. Obstet Gynecol.

[32] Alaszewski A, Horlick-Jones T (2003). How can doctors communicate information about risk more effectively?. BMJ.

[33] Simmons HA, Goldberg LS (2011). High-risk pregnancy after perinatal loss: understanding the label. Midwifery.

[34] Taghrir MH, Borazjani R, Shiraly R (2020). COVID-19 and Iranian medical students; a survey on their related-knowledge, preventive behaviors and risk perception. Arch Iran Med.

[35] Aenishaenslin C, Michel P, Ravel A (2015). Factors associated with preventive behaviors regarding Lyme disease in Canada and Switzerland: a comparative study. BMC Public Health.

